# The Abortive Treatment of Acute Pelvic Inflammation

**Published:** 1889-12

**Authors:** Virgil O. Hardon

**Affiliations:** Professor of Obstetrics and Diseases of Women and Children, Atlanta Medical College, Atlanta, Ga.


VOL. VI. DECEMBER, 1889. NO. 10.

### Original Communications.

### THE ABORTIVE TREATMENT OF ACUTE PELVIC INFLAMMATIONS.*

BY VIRGIL O. HARDON, M. D.,

Professor of Obstetrics and Diseases of Women and Children, Atlanta Medical College, Atlanta, Ga.

The views held by gynecologists in regard to acute inflammation in the female pelvis have, within the past few years, undergone a radical change. The inflammatory processes formerly regarded as idiopathic, or, at least, as primary affections, are now almost universally recognized as dependent upon antecedent disease in the ovaries or fallopian tubes, especially the latter. This change of opinion has been, to a very large extent, the result of advances in abdominal surgery, which have enabled the conditions of the pelvic organs to be studied in the living subject, by immediate inspection and palpation, instead of through

*Read before the Southern Surgical and Gynecological Association, at Nashville, Tenn., November 12th, 1889.

the medium of the vaginal tissues. Consequently, it is found that the change of opinion on this subject has been most marked among those men who have had large experience in abdominal work. The diagnostic value of abdominal section, under such circumstances, is almost as great as that of *post mortem* examination, and hence the opinion of the laparotomist is entitled to very great weight. As a result of this mode of observation, the conclusion cannot be avoided that acute pelvic inflammation is, at least in the majority of cases, associated with septic, gonorrhœal, tubercular, or some other form of inflammation of the tubes.

The acceptance of this theory has led to the adoption of a line of treatment which has proven its correctness, and which has produced results often brilliant, and nearly always satisfactory to both physician and patient. This treatment consists of the removal of the diseased organs by laparotomy; such removal being usually followed by a cessation of the inflammatory process, and a speedy and permanent restoration to health. This treatment so commends itself to the reason upon theoretical grounds, and is followed by so excellent clinical results, that it may truly be said that he who rejects it carries conservatism to a fault, and is not open to conviction.

But unfortunately, it is not always practicable to adopt this line of treatment. In the first place, such attacks usually occur suddenly, and the patient comes under the care of her family physician, a general practitioner, it may be, who possesses neither the courage nor the skill to attempt a laparotomy. The patient may be too far from a city to obtain the services of a specialist, or her pecuniary condition may be such as to render that course impossible. To her, therefore, this door to relief is practically closed. In the second place, the announcement to the patient that an abdominal section offers the only means of cure often falls with the startling effect of thunder from a clear sky. The magnitude of the operation appears to her entirely out of proportion to the gravity of the affection, and she decides that the remedy is worse than the disease. If the operation is urged she passes from observation, and seeks the aid of a physician who is more willing to accommodate his treatment to the views of the patient. Moreover, even though the physician be a skilled laparotomist, and the consent of the patient be gained, the period of election for such an operation is not at the height of an acute inflammatory attack, but rather after the violence of the inflammation has abated. It often happens, therefore, that the physician is constrained to adopt a line of treatment less radical and less rational, and which shall be simply palliative in its nature.

But under such circumstances he is at once confronted by the well recognized fact, that to allow the inflammatory process to pursue its natural tendency to self-limitation is to expose the patient to sequelæ which may be more disastrous than the acute inflammation itself. In the majority of cases inflammatory exudations are thrown out which persist more or less permanently, producing distortions, constrictions and obstructions of the pelvic organs, interfering with their vitality and their functions, and jeopardizing comfort, health, and even life. If an operation be ultimately determined upon, it will often be found that these exudates have so matted all the parts together, so altered their relations and changed their physical conditions, that the recognition of the organs to be removed is rendered impossible, or, if they are recognized, their removal cannot be accomplished.

For these reasons an obvious and important *desideratum* is the discovery of some method by which acute pelvic inflammations may be shorn of their dangerous features without resort to laparotomy. If the exudation into the pelvic tissues can be prevented, pelvic inflammations will have become comparatively trivial and innocuous affections, and will be of importance only as indications of more serious disease in the tubes, of which the inflammatory attacks are but unimportant incidents. To show how this object can be accomplished shall be the purpose of this paper.

There are two forms of inflammation which are recognized as occurring within the pelvis as a result of tubal disease, pelvic cellulitis and pelvic peritonitis. In many cases both forms of inflammation are simultaneously present in the same subject, and it is not improbable that in all cases when either is present, the other also exists in a greater or less degree. It is hardly possible, when one considers the anatomical relations of the pelvic cellular tissues to the peritoneum, to conceive of an inflammation of the one structure without a greater or less implication of the other. But in the majority of cases one form of inflammation can be recognized as forming the preponderating element of the the disease, to which the other plays only a secondary *role.* The two affections are accordingly distinguishable to some extent by their symptomatology and to a greater extent by the signs presented upon physical examination.

Although some authors strenuously insist that "pelvic cellulitis is practically a myth," yet the best authorities recognize the possibility of its existence in connection with tubal disease. Its occurrence in the acute form has been demonstrated both by abdominal section, and by *post mortem* examination. It has a characteristic clinical history and presents physical phenomena which are not found in acute pelvic peritonitis or acute salpingitis when occurring alone. The course of the disease presents three stages, viz., a stage of effusion in which serum from the blood is poured out into the meshes of the pelvic cellular tissue; a stage of solidification in which the cellular tissue is filled with a solid exudate of plastic lymph; and a final stage in which this exudate either undergoes absorption or breaks down into pus. These three stages are recognizable clinically. The stage of effusion is of short duration, usually lasting from twenty-four to forty-eight hours. During this stage a vaginal examination will show the roof of the vagina to possess a boggy, œdematous feeling, and to be so swollen that it is almost or quite level with the external os, so that the cervix feels as if it were drawn up into the surrounding tissues. This swelling may be found behind the uterus, upon one or both sides, or in all three localities. The uterus is still movable, but its movements give great pain.

After a period of from twenty-four to forty-eight hours the swelling subsides and the portions of the roof in which it existed will be found filled with a solid exudate which is hard and unyielding to the touch, and which effectually destroys the mobility of the womb. This exudate is after a time gradually absorbed or else breaks down into pus, forming a pelvic abscess.

Embedded within the exudate lie the ovaries, the tubes and the blood vessels, lymphatics and nerves of the pelvic organs. The cellular tissue undergoes cicatricial contraction as the exudate is absorbed, and such contraction draws the uterus from its normal position, constricts the ovaries, causing atrophy and degeneration, occludes the tubes at one or more points, interfering with the escape of their secretions by their natural channel through the uterus, and deranges the circulation, the nutrition and the innervation of the pelvic organs, with impairment of all their functions.

The constitutional symptoms consist of acceleration of the pulse, elevation of temperature, severe pain in the pelvis, extending down the thighs, tenderness of the hypogastric and inguinal regions, a sense of weight and fulness in the pelvis, difficult micturition and painful defecation. These symptoms are most marked in the first stage, and are somewhat diminished as soon as solidification has taken place. They continue, however, with lessened intensity far into the third stage of the disease.

On account of the brief duration of the first stage it often happens that the patient is not seen by the physician until solidification has taken place. It is unfortunate that this is so, since it is only during the stage of effusion that treatment can be successfully applied for aborting the inflammation. If the effusion into the pelvic cellular tissue can be gotten rid of, the disease is cut short, the second stage does not occur and after a brief convalescence the patient is restored to her usual health, with her pelvic tissues in as good condition as before the occurrence of the attack. The opportunity for the adoption of a line of treatment which will accomplish this end occurs most often in private practice, where the patient comes under the care of the physician at the commencement of the attack.

The treatment consists in the withdrawal of the effusion from the cellular tissue by means of the aspirator. It is accomplished in the following manner: The patient is placed across the bed in the dorsal position with the hips near the edge and the legs flexed. The vagina is syringed out with a warm antiseptic solution, and the hands and the aspirator are also rendered thoroughly aseptic. A finger introduced into the vagina locates the effusion by the œdema of the vaginal roof, and also serves as a guide for the introduction of the needle. The instrument which I use is an ordinary exploring aspirator. The needle is thrust into the cellular tissue to a distance of about half an inch and the piston withdrawn until the fluid ceases to flow. The needle is then reinserted at successive points until the swelling and œdema are felt to have disappeared. I have found that three punctures on each side of the uterus are usually sufficient, and if the effusion is present behind the cervix, one or two additional punctures in that locality are also required. The fluid which is withdrawn is a bloody serum, varying in quantity from one to two fluid drachms at each puncture. As a rule it is not necessary to anæsthetize the patient, although I have sometimes done so on account of extreme nervousness and dread of the operation. The pain is not severe.

The relief which follows the withdrawal of the fluid is immediate. The patient usually falls into a quiet sleep, which is in marked contrast to the pain and restlessness which had previously existed. The pulse and temperature rapidly decline, the acute pain gives place to moderate tenderness upon pressure, the constitutional symptoms disappear, and in forty-eight hours the patient feels as well as before the commencement of the attack. The most gratifying result of this treatment, however, is found in the fact that subsequent vaginal examination will show that no solidified plastic lymph remains in the pelvic cellular tissue to give rise to troublesome sequelæ. A slight thickening at the site of the former œdema is all that is left as a reminder of the acute inflammation. If, as is usually the case, disease of the tubes had previously been present, this condition is still found to exist. The effect of the aspiration is simply to cut short the acute inflammatory attacks and not to remove or even palliate its cause.

In a correspondence upon this subject with Dr. J. M. Baldy, of Philadelphia, he suggested to me that the use of Epsom salts, in sufficient quantity to induce free catharsis, would have the same effect as aspiration in aborting this disease. I have tested the value of this suggestion in a sufficient number of cases to convince me that, while the use of salts controls to a very great extent the pain and constitutional symptoms, it does not prevent the deposit and solidification of plastic lymph in the cellular tissue. It is therefore much inferior in value to the use of the aspirator.

The second form of pelvic inflammation which may be associated with disease of the tubes, and which is much more frequently met with under such circumstances than pelvic cellulitis, is pelvic peritonitis. The constitutional symptoms in this affection are very similar to those of cellulitis, since in both they are the result of the inflammatory process. In peritonitis, however, there is usually a greater amount of nausea and prostration. By physical examination the diagnosis may be more conclusively established. The finger in the vagina will detect a thickening in the tissues of the vaginal roof, with excessive pain upon pressure. There is, however, in this affection an absence of the boggy, œdematous feeling which is so prominent a feature of pelvic cellulitis. In fact the absence of this condition is almost the only decisive diagnostic point of distinction between the two varieties of inflammation. In pelvic peritonitis there is undoubtedly a serous exudation into the peritoneum which gravitates into the pelvis. But it is impossible to detect such fluid when it is free in the peritoneal sac. Lymph is thrown out upon the serous surfaces of the pelvic viscera by which they are agglutinated together, producing adhesions which subsequently undergo contraction, forming bands which may often be detected by the finger in the later stages of the disease. Or these adhesions may result in the matting together of all the pelvic viscera into an inextricable mass. The results of such agglutination are disastrous in the extreme, impairing function, deranging circulation and innervation and causing pain by constriction and pressure. The presence of such adhesions also forms a serious obstacle to the removal of the diseased tubes, should such a procedure be subsequently determined upon. Hence to abort the disease and prevent the development of such untoward sequelæ becomes a matter of as great importance in pelvic peritonitis as in pelvic cellulitis.

The adhesions which occur in pelvic peritonitis are due to the lymph which is thrown out by opposing serous surfaces. The removal of such lymph by mechanical means is an obvious impossibility. To limit its formation as far as possible by cutting short the inflammatory process is all that can be hoped for. My experience has proven that this can be accomplished. Taking a hint from the suggestion of Dr. Baldy, referred to above, and also from my experience in the treatment of the acute peritonitis which sometimes follows abdominal section, I have, during the past year, been treating all cases of acute pelvic peritonitis which have come into my hands in the early stages by active catharsis.

As soon as the disease is recognized, I commence to give teaspoonful doses of Epsom salts every hour, dissolved in water as hot as can be swallowed, until watery stools are induced. From five to eight doses are usually required, and are followed by several copious evacuations in rapid succession. If the patient has previously taken an opiate for the relief of pain, a larger number of doses is necessary. As soon as free catharsis takes place, the pulse and temperature begin to decline, and within twelve hours the fever is abated, the pain is relieved, the nausea and prostration disappear, and the patient goes on to a rapid convalescence. The relief is so immediate and so marked, that the patient fully recognizes its dependence upon the treatment, and I have a number of patients who are subject to recurrent attacks of pelvic peritonitis from diseased tubes, who have learned to abort such attacks by immediately resorting to Epsom salts without calling in a physician.

I have found that successive attacks, when cut short in this way, do not lead to an increase of the amount of plastic deposit in the pelvis, but on the other hand, the depletion which follows the use of the remedy seems to diminish such deposits. By this fact I have been led to adopt the plan of giving to my patients frequent repetitions of this course of treatment in the intervals between the acute attacks, with the effect of producing a more rapid absorption of such deposits than I have been able to accomplish by any other method.

I could cite a large number of instances in which I have successfully used these two methods of treatment. I will, however, report only two cases, for the purpose of illustration :

CASE I. PELVIC CELLULITIS TREATED BY ASPIRATION.—Mrs. N. W.; nullipara ; has always enjoyed good health until her present attack. On the 29th of September, 1887, after a long and fatiguing walk, she was seized with pain in the pelvis, most marked on the left side and extending down into the left thigh. During the night the pain rapidly grew worse, so that she was unable to sleep. Micturition became extremely painful, and an action of the bowels, which occurred during the night, gave her great distress. The abdomen became excessively tender and high fever developed. I saw her on the following day and found her tossing in bed and moaning with pain. The face was flushed, the skin hot and dry, temperature 102.6°, pulse 136. She complained of pain throughout the whole pelvis, but particularly in the left groin and hip. Examination showed the vagina to be hot and dry. On the left side of the cervix the vagina was œdematous and swollen, and the condition extended behind the cervix into Douglas' pouch. The uterus was pushed over toward the right side of the pelvis. The womb was movable, but its movement gave great pain. The pelvis was everywhere very sensitive to pressure. With strict antiseptic precautions an aspirator needle was thrust into the cellular tissue at four successive points, and about six drachms of bloody serum withdrawn. The patient at once experienced relief and soon after sank into a quiet sleep, which lasted nearly three hours. When I saw her six hours later the temperature had fallen to 99.4° and the pulse to 94, and she was almost entirely free from pain. She slept all night without an anodyne, and the following day her pulse and temperature were normal and she was anxious to get up. I enjoined quiet for several days, but on the third day after the aspiration I met her on the street and she declared that she felt as well as she ever did in her life. Two weeks afterwards I made a vaginal examination and found no trace of hardness in the roof of the vagina and perfect mobility of the uterus. The left fallopian tube could be felt to be enlarged, but was not tender to pressure. I have seen the patient at intervals since, during a period of two years, and she has had no recurrence of the inflammation in that time.

CASE II. PELVIC PERITONITIS TREATED BY ACTIVE CATHARSIS.—Mrs. W. R. H., age twenty-eight, has borne two children at term. In June, 1889, she aborted at about two months with excessive hemorrhage, but made an apparently good recovery. On the 21st of July, while engaged in domestic duties, she was seized with severe pain in the pelvis, which rapidly increased, with the development of high fever, thirst, nausea and great tenderness of the abdomen. Micturition was painful, the bowels constipated. I saw her the following morning. She was lying on her back with her knees drawn up, and the expression of her face denoted intense suffering. The abdomen was tympanitic and very tender to the slightest touch, especially in the lower portion. She had vomited during the night and was still nauseated. Temperature, 103.2°, pulse 142. By vaginal examination the whole pelvis was so sensitive that little could be determined beyond the fact that no œdema or swelling existed in the roof of the vagina, and the womb was somewhat movable. The slightest pressure caused her to cry out with agony. I at once gave her a teaspoonful of Epsom salts in hot water, which was repeated every hour until eight doses had been taken. By the following morning she had eleven watery evacuations, some copious and others scanty. When I saw her on the next day the temperature had fallen to 99.8°, and the pulse to 102. The tympanites had disappeared and the pain nearly so. The tenderness had notably diminished, so that I was able to make a satisfactory examination and to determine the existence of a double pyosalpinx. She made a rapid convalescence without further treatment beyond keeping the bowels moderately loose by occasional doses of salts, and in five days was able to resume the duties of her household with perfect ease and comfort. In September, during my absence at the North, she had a second attack similar to the one just described. At the commencement of the pain she resorted to the same remedy which I had prescribed, and the disease was aborted in a few hours.

